# Supplementary material for: Hyaluronan synthases; mechanisms, myths, & mysteries of three types of unique bifunctional glycosyltransferases
Source: Glycobiology. 2023 Sep 28;33(12):1117–27. doi: 10.1093/glycob/cwad075 (PMC10939387; doi:10.1093/glycob/cwad075)
Supplement: DeA_and_Zimm_HAS_Review_91423_REDLINE_cwad075 [file dea_and_zimm_has_review_91423_redline_cwad075.pdf]

"Hyaluronan synthases; mechanisms, myths, & mysteries of three types of unique bifunctional glycosyltransferases"

Paul L. DeAngelis[1] and Jochen Zimmer[2]

[1] Dept. of Biochemistry and Molecular Biology, University of Oklahoma Health Sciences Center; 940 Stanton L. Young Blvd., Oklahoma, OK 73104, USA

[2] Howard Hughes Medical Institute and Dept. of Molecular Physiology & Biological Physics, University of Virginia, 480 Ray C. Hunt Dr., Charlottesville, VA 22908, USA

Corresponding authors: paul-deangelis@ouhsc.edu ; jz3x@virginia.edu

Running Head: Hyaluronan Synthases

Keywords: biosynthesis/catalysis/enzyme/polymerization/polysaccharide

Abstract

Hyaluronan (HA), the essential [-3-GlcNAc-1-β-4-GlcA-1-β-]$_n$ matrix polysaccharide in vertebrates and molecular camouflage coating in select pathogens, is polymerized by "HA synthase" (HAS) enzymes. The first HAS identified three decades ago opened the window for new insights and biotechnological tools. This review discusses current understanding of HA biosynthesis, its biotechnological utility, and addresses some misconceptions in the literature.

HASs are fascinating enzymes that polymerize two different UDP-activated sugars via different glycosidic linkages. Therefore, these catalysts were the first examples to break the "one enzyme/one sugar transferred" dogma. Three distinct types of these bifunctional glycosyltransferases (GTs) with disparate architectures and reaction modes are known. Based on biochemical and structural work, we present an updated classification system. Class I membrane-integrated HASs employ a processive chain elongation mechanism and secrete HA across the plasma membrane. This complex operation is accomplished by functionally integrating a cytosolic catalytic domain with a channel-forming transmembrane region. Class I enzymes, containing a single GT family-2 (GT-2) module that adds both monosaccharide units to the nascent chain, are further subdivided into two groups that construct the polymer with opposite molecular directionalities: Class I-R and I-NR elongate the HA polysaccharide at either the reducing or the non-reducing end, respectively. In contrast, Class II HASs are membrane-associated peripheral synthases with a non-processive, non-reducing end elongation mechanism using two independent GT-2 modules (one for each type of monosaccharide) and require a separate secretion system for HA export. We discuss recent mechanistic insights into HA biosynthesis that promise biotechnological benefits and exciting engineering approaches.

Introduction

Hyaluronan (hyaluronic acid, hyaluronate, HA) is an essential polysaccharide in the extracellular matrix of mammals and found in all chordates from the *Amphioxus* lancet to humans (Fig. 1). This 'wonder goo' plays many roles in development, health, and disease as covered by multiple reviews (Itano, N., Zhuo, L., et al. 2008, Simpson, M., Schaefer, L., et al. 2022, Tammi, M.I., Day, A.J., et al. 2002, Toole, B.P. 2000) with more physiological functions being reported almost monthly. Interestingly, certain microbial animal pathogens also employ an extracellular HA coating (capsule) as a virulence factor; this 'self' molecule appears to serve as molecular camouflage to dampen host immune defenses (Guan, L., Zhang, L., et al. 2020, Wessels, M.R. 2019). In the last three decades, we have made strides from the identification of various HA biosynthesis enzymes, the HA synthases (HASs), to a better understanding of their structure and function (Weigel, P.H. and DeAngelis, P.L. 2007). Recent studies provide the first structural snapshots of a HAS at different stages during HA biosynthesis (Maloney, F.P., Kuklewicz, J., et al. 2022). This review presents an updated classification system for HASs, discusses reaction mechanisms and biotechnological utility, and points out some potential technical misconceptions as well as areas ripe for future investigations.

HA Synthase Basics

General HAS Reaction: HASs belong to glycosyltransferase (GT) family-2 (GT-2), a huge conglomeration of glycan-building enzymes, including cellulose, chitin and glycogen synthases as well as several Golgi localized glycoprotein *N*-glycan transferases (Lombard, V., Ramulu, H.G., et al. 2014). HASs synthesize the [-3-GlcNAc-1-β-4-GlcA-1-β-]$_n$ disaccharide repeats of the HA polysaccharide by repetitive addition of the monosaccharide units from uridine diphosphate-activated (UDP) donors (Fig. 1) (Weigel, P.H. and DeAngelis, P.L. 2007). This

reaction releases UDP as a second reaction product, which has been shown to competitively inhibit some GT-2s *in vitro* (Omadjela, O., Narahari, A., et al. 2013, Purushotham, P., Cho, S.H., et al. 2016, Tlapak-Simmons, V.L., Baron, C.A., et al. 2004). Both UDP-*N*-acetylglucosamine (GlcNAc) and UDP-glucuronic acid (GlcA) precursors are present in the cytoplasm; their levels and interplay with the synthetic machinery may define the amount and size of the final HA product formed (Zimmer, B.M., Barycki, J.J., et al. 2022).

In general, Class I enzymes are integrated into the plasma membrane whereas Class II HAS, so far only been identified in Gram-negative bacteria, associates with the cytosolic side of the inner membrane (Jing, W. and DeAngelis, P.L. 2000). Class I HASs are found in prokaryotes (Gram-positive Group A and C *Streptococci*), a *Chlorella* virus, and vertebrates. This group is further classified based on the chain elongation mechanism, as described below (Fig. 1).

HA is an extracellular polysaccharide aiding in extracellular matrix formation (animals) or encapsulation (microbes, virally infected hosts). It is noteworthy that, depending on species and exact tissue, HA chains ranging in length from ~10 kDa to 10 MDa (~25 to 25,000 sugar repeats) are efficiently synthesized and exported during or after biosynthesis.

The known HAS enzymes require divalent metals (e.g., magnesium or manganese) for activity, with the specific cation preference being synthase dependent (Blackburn, M.R., Hubbard, C., et al. 2018, DeAngelis, P., Jing, W., et al. 1997, Kumari, K. and Weigel, P.H. 1997, Pummill, P.E., Achyuthan, A.M., et al. 1998, Tlapak-Simmons, V.L., Baron, C.A., et al. 2004). As in many types of GTs, the cation complexes the diphosphate group of the UDP-sugar substrates, facilitating transition state formation and/or providing leaving group assistance. A conserved Asp-X-Asp motif contributes to metal coordination. Of note, recent work on the viral HAS revealed a second Mn^{2+}-binding site also in proximity to the nucleotide's diphosphate, and

mutagenesis experiments demonstrated its importance for catalytic activity, for hitherto unknown reasons (Maloney, F.P., Kuklewicz, J., et al. 2022).

In all known HASs, it appears that only one sugar unit is transferred to the nascent chain at a time (Fig. 2); this fact has been shown for PmHAS (DeAngelis, P.L. 1999) and viral HAS (Maloney, F.P., Kuklewicz, J., et al. 2022) and agrees well with the chain elongation mechanism employed by cellulose synthase (Morgan, J.L., McNamara, J.T., et al. 2016). We speculate that this mechanism also applies to the rest of the HAS enzymes. This model is in contrast to some earlier hypotheses on cellulose and HA biosynthesis that assumed that the glycosyl units of the disaccharide units were added simultaneously (Weigel, P.H. 2015) (Carpita, N.C. 2011, Saxena, I.M., Brown, R.M., et al. 2001).

The overall reaction catalyzed by all known HASs is shown in Equations 1 or 2. The official IUBMB enzyme classification for a HAS is EC 2.4.1.212; however, depending on the particular enzyme involved, there are multiple routes to make the same identical HA polysaccharide.

Equation 1

$$n\ \text{UDP-GlcNAc} + n\ \text{UDP-GlcA} \xrightarrow[\text{Mg}^{2+},\ \text{Mn}^{2+}]{\textit{HAS}\ \textbf{(Class I-NR, Class II)}} 2n\ \text{UDP} + [\text{GlcNAc-GlcA}]_n = \text{HA}$$

Equation 2

$$n\ \text{UDP-GlcNAc} + n\ \text{UDP-GlcA} \xrightarrow[\text{Mg}^{2+},\ \text{Mn}^{2+}]{\textit{HAS}\ \textbf{(Class I-R)}} 2n\text{-1 UDP} + \text{UDP-}[\text{GlcNAc-GlcA}]_n$$

HA is quite unlike the other structurally similar glycosaminoglycans found in vertebrates, heparan sulfate/heparin, chondroitin sulfate, and keratan sulfate (Pedersen, L.C., Tsuchida, K., et al. 2000). This disparity is because HA is neither made in the Golgi apparatus on a protein core (using different types of peripheral membrane GTs), nor is the chain modified by sulfation or epimerization in post-polymerization processes.

HAS Nomenclature: The various HAS proteins are named in a fashion akin to DNA-acting enzymes; the first letters of the genera and species are employed to denote the source organism. Thus, the synthase from *Streptococcus pyogenes* is SpHAS, and a human enzyme is HsHAS. In the latter case, there are three isozymes named HAS1, 2 and 3, but some extant chordates have four genes (some may be pseudogenes) based on ancestral repeated gene duplication events. One of the initial vertebrate HASs to be identified from the frog *Xenopus laevis* (DeAngelis, P.L. and Achyuthan, A.M. 1996, Meyer, M.F. and Kreil, G. 1996) was initially named DG42 (for an RNA differentially expressed during gastrulation) (Sargent, T.D., Jamrich, M., et al. 1986), but formally would be called XlHAS1.

Enzyme Sequence Identification: The HA polysaccharide was found to be abundant in certain vertebrate tissues (notably, the seminal discovery in the vitreous humor of mammalian eye by Karl Meyer in the 1930s (Meyer, K. and Palmer, J. 1936)) and the surface coating of Group A and C *Streptococci* (Kendall, F.E., Heidelberger, M., et al. 1937). The 'synthase' ('synthetase' or 'polymerase' in some older nomenclatures) activity that generated HA was initially studied with radiolabeled sugar incorporation assays of crude membrane preparations by Albert Dorfman and colleagues starting in the mid-1950s (Markovitz, A., Cifonelli, J.A., et al. 1959). These pioneering biochemical experiments laid the groundwork for generating Equation 1 and spawned the first mechanistic hypothesis on HA chain elongation in 1959 (Fig. 2A).

The primary sequences of the first HAS enzymes were identified in 1993-1998 by combinations of molecular and genetic methodologies (e.g., transposon insertional mutagenesis, expression cloning, degenerate polymerase chain reaction) because these low abundance membrane proteins were rather recalcitrant to classical analyses (DeAngelis, P.L., Jing, W., et al. 1998, DeAngelis, P.L., Papaconstantinou, J., et al. 1993, Itano, N. and Kimata, K. 1996, Spicer, A.P., Augustine, M.L., et al. 1996). Indeed, the early reports using biochemical purification, affinity labeling and/or immunoreagents mis-identified (Lansing, M., Lellig, S., et al. 1993, Prehm, P. 1989) (Mian, N. 1986) or missed pinpointing (van de Rijn, I. and Drake, R.R. 1992) the authentic HASs.

Three Distinct Types of HA Synthases

A generalized overview of HASs: All known, experimentally confirmed HA synthases are bifunctional GTs that add both the GlcA and GlcNAc components to the nascent HA chain. These findings broke the longstanding glycobiology dogma that 'one enzyme transfers one type of sugar and forms one type of linkage' (DeAngelis, P.L. 1999, Weigel, P.H. and DeAngelis, P.L. 2007); it was assumed that a pair of GT enzymes (i.e. a separate polypeptide for each monosaccharide unit) would be necessary to form the disaccharide repeats of HA. Following their discoveries, it became apparent that the HASs from different organisms did not always appear or behave equivalently. Accordingly, biochemical and structural features of these unique enzymes were employed to assign the various HASs into three main types (Weigel, P.H. and DeAngelis, P.L. 2007).

Features of the streptococcal HASs: The original prototype HAS from the Gram-positive *Streptococcus pyogenes,* SpHAS (encoded by the *hasA* gene), was shown to be a single

polypeptide species with one identifiable GT domain that alternates between adding GlcA or GlcNAc units (Blackburn, M.R., Hubbard, C., et al. 2018, DeAngelis, P.L., Papaconstantinou, J., et al. 1993), but the details of modulating the UDP-sugar binding and elongation reaction are still unclear. The streptococcal HAS is a membrane-integrated protein (predicted to have 4 transmembrane (TM) segments) that also secretes HA across the plasma membrane (Heldermon, C., DeAngelis, P.L., et al. 2001, Hubbard, C., McNamara, J., et al. 2012).

The use of disparate, native enzyme sources in the past gave conflicting answers regarding the general elongation mechanism for the HASs. *Streptococcus* HAS elongated HA on its reducing end (Class I-R) based on pulse-chase labeling reactions with an *Escherichia coli*-derived recombinant enzyme as dissected by exoglycosidase digestion time courses (Tlapak-Simmons, V.L., Baron, C.A., et al. 2005). This method was further refined by the use of recombinant SpHAS (as well as XlHAS1 for a parallel comparison) expressed in *Saccharomyces cerevisiae*, a heterologous expression host lacking HA as well as the UDP-GlcA substrate (Bodevin-Authelet, S., Kusche-Gullberg, M., et al. 2005). Here, HA biosynthesis could be cleanly initiated *in vitro* without any background. Other support is derived from mass spectral analyses of early reaction products during synthesis *in vitro* with purified recombinant SeHAS (*Streptococcus equisimilis*) enzyme (Weigel, P.H., West, C.M., et al. 2015). Further, in contrast to non-reducing end polymerization, HA elongation at the reducing end requires its activation by covalent coupling to a nucleotide (Eq. 2). Indeed, using isotope labeling, the SeHAS-produced HA polymer was shown to be covalently attached to UDP, thereby supporting the reducing-end elongation mechanism (Blackburn, M.R., Hubbard, C., et al. 2018, Tlapak-Simmons, V.L., Baron, C.A., et al. 2005).

Chitin oligosaccharides ([-4-GlcNAc-1-β-]$_n$ where n = ~2-6) fused onto HA chains as a non-reducing end cap were also reported (Weigel, P.H., West, C.M., et al. 2015). HA derived from native *Streptococcus* that was digested with hyaluronidase also appeared to have chitin caps at the non-reducing termini. These findings imply that (*i*) UDP-GlcNAc is the initial acceptor for SeHAS, (*ii*) this group is extended by sequential addition of several GlcNAc units in the early stages, and then (*iii*) followed by disaccharide repeat addition to make the very large HA chain. The role(s) of the theorized chitin cap *in vivo* remains unknown and so is the molecular cue that switches from chitin to HA biosynthesis. Several functions of a chitin cap have been discussed, including serving as a plug in the membrane to prevent ion leakage during HA synthase maturation or participating in interactions with other proteins (Weigel, P.H. 2015). Some controversy also exists on whether vertebrate HASs can make chitin as well (Varki, A. 1996). At this point, it is unclear whether the proposed chitin cap is indeed necessary for *in vivo* HA biosynthesis or may be the result of particular experimental conditions.

Additional insights into the Class I-R reaction mechanism came from biochemical and biophysical analyses of SeHAS (Blackburn, M.R., Hubbard, C., et al. 2018). Initially, streptococcal enzymes were hypothesized to function as monomers based on radiation inactivation analysis (Tlapak-Simmons, V.L., Kempner, E.S., et al. 1998); the actual target size reported was ~50% larger than the predicted polypeptide (but less than the postulated dimer), therefore, a complex of the protein monomer with ~16 'essential lipid' molecules was invoked in their model. Later, single particle photobleaching and biochemical experiments showed that the SeHAS enzyme functions as a homodimer (Blackburn, M.R., Hubbard, C., et al. 2018). This finding was supported by the purification of a mixed complex of wildtype and inactive mutant SeHAS, which lacked catalytic activity, thus suggesting that an SeHAS homodimer is the

biolofically functional unit. According to this updated model, it is predicted that the GT domains of a dimer would bind the UDP-attached HA polymer as well as the UDP-sugar substrate (Fig. 2B).

Although initially proposed under the assumption of a monomeric functional polypeptide unit, the “pendulum hypothesis” (Weigel, P.H. 2015) agrees well with the above-described dimeric functional unit of Class I-R enzymes. One protomer would interact and position the UDP-attached reducing end of HA (see Eq. 2), while the other binds the incoming UDP-donor substrate. Glycosyl transfer would than attach the nascent HA chain to the substrate molecule, such that the HA-attached UDP originates from the last incorporated substrate. According to this reaction scheme, the nascent HA chain functions as the donor and the substrate molecule as the acceptor of the glycosyl transfer reaction (Fig. 2B). Further information derived from structural biology will be required to fully elucidate the molecular details of the Class I-R HASs.

Features of the vertebrate and viral HASs: Within several years of the SpHAS discovery, the vertebrate (HAS1,2,3 isozymes (Weigel, P.H., Hascall, V.C., et al. 1997)) and viral (CvHAS of *Paramecium bursaria Chlorella* virus 1, PBCV-1 (DeAngelis, P.L., Jing, W., et al. 1997)) HASs were identified. The vertebrate HASs produce HA of the glycocalyx and extracellular matrix. HA is essential in mammals. Identifying the viral enzyme as an authentic HAS was the first GT found to be encoded by a virus and the first instance of this ‘animal’ polymer in a plant-like organism.

These enzymes display similarities in overall TM architecture, with two TM helices on the N-terminal and four on the C-terminal side of the cytosolic GT domain (Fig. 2B and 3). The animal and viral HASs share the features of a single GT-2 catalytic domain that operates as in Eq. 1, but their reaction mechanism is inverted compared to streptococcal HAS (Eq. 2). Here,

new sugar units are added to the non-reducing (NR) end of the nascent chain (Fig. 2B) (Blackburn, M.R., Hubbard, C., et al. 2018, Hoshi, H., Nakagawa, H., et al. 2004, Maloney, F.P., Kuklewicz, J., et al. 2022), thus these catalysts are termed Class I-NR HASs.

This NR end reaction mode is typical for the vast majority of known GT enzymes and contrasts the reducing-end elongation mechanism employed by streptococcal HASs described above. The major difference is that in NR end elongation, the nascent HA chain serves as the acceptor of the transfer reaction by mediating a nucleophilic attack on the substrate's donor sugar.

Recent cryo electron microscopy (EM) and functional analyses of viral HAS provided new insights into the initiation stage of HA biosynthesis (Fig. 3) (Maloney, F.P., Kuklewicz, J., et al. 2022). First, it was shown that the enzyme's GT domain tightly interacts with a channel-forming TM region to couple HA synthesis with secretion. Second, biochemical and structural data revealed that HA biosynthesis starts from a GlcNAc monosaccharide primer hydrolytically released from the substrate UDP-GlcNAc. GlcA, however, does not initiate biosynthesis. Third, because the enzyme's catalytic pocket and TM channel are juxtaposed, HAS can 'hold on' to the nascent polysaccharide between elongation steps, thereby enabling processive chain elongation.

Another important finding from these cryo EM studies is that the viral enzyme functions as a monomer. Despite using large amounts of purified enzyme for these studies, no evidence of homo-oligomerization was observed, in contrast to trimerization of plant cellulose synthases (Purushotham, P., Ho, R., et al. 2020). This model is in agreement with the previous radiation inactivation data on XlHAS1 (Pummill, P.E., Kempner, E.S., et al. 2001); in these studies, the comparison of native and fusion protein constructs allowed internal calibration of the functional target size as a monomer. However, recombinantly over-expressed human HASs were reported

to form homo- (HAS2-HAS2) and heterooligomers (HAS1-HAS2 and HAS3-HAS1) by FRET imaging and immunoprecipitation assays (Bart, G., Vico, N.O., et al. 2015). Therefore, more studies are needed to identify the presence and physiological relevance of HAS oligomers during HA biosynthesis *in vivo* in various species.

Class I HASs form an HA secretion channel. Based on the predicted transmembrane segments of SpHAS and the observation that a single gene conferred HA production, it was hypothesized that the enzyme could transport HA through a membrane pore (Heldermon C., DeAngelis P.L., Weigel P.H., 2001). *In vitro* HA biosynthesis assays with proteoliposome reconstituted enzymes demonstrated that SeHAS and CvHAS are both necessary and sufficient to translocate HA across the vesicle membrane during synthesis (Blackburn, M.R., Hubbard, C., et al. 2018, Hubbard, C., McNamara, J., et al. 2012). Structural analyses of CvHAS revealed that the enzyme creates a continuous TM channel upon priming with a GlcNAc monosaccharide (Maloney, F.P., Kuklewicz, J., et al. 2022). The channel is electropositive and lined with several polar residues that could contact HA during secretion. In an extended conformation, about ten glycosyl units, i.e., five HA disaccharide repeat units, would span the TM channel (Fig. 3).

HA likely adopts multiple conformations in an aqueous environment such as the extracellular matrix. Spectroscopic analyses, modeling, and high-resolution structural insights showed when bound to some proteins, the acetamido and carboxylate groups of an HA repeat unit are positioned on the same side of the disaccharide (Almond, A., DeAngelis, P.L., et al. 2006, Banerji, S., Wright, A.J., et al. 2007). How the polymer chain migrates through HAS's TM channel is unknown to date. However, the CvHAS channel is sufficiently wide to allow conformational flexibility during translocation. Further high-resolution structural insights into HA translocation intermediates are necessary to determine the precise translocation mechanism.

A revised HA biosynthesis model for Class I-NR enzymes: The structural and functional analyses of CvHAS suggest a revised working model for HA biosynthesis (Fig. 4). First, the enzyme initiates HA biosynthesis by binding and hydrolyzing a UDP-GlcNAc substrate molecule. The released GlcNAc monosaccharide is stabilized at the acceptor binding site near the entrance to a TM channel. The primer's acetamido group ensures the selectivity of the catalytic pocket for UDP-GlcA, leading to the formation of a GlcNAc-GlcA disaccharide. Prior or concomitant to binding of the next substrate molecule (UDP-GlcNAc), the disaccharide unit has to translocate into HAS's TM channel to position GlcA at the acceptor site. In this position, GlcA's carboxylate group likely prevents binding of a UDP-GlcA substrate molecule. The process of stepwise elongation and translocation continues until HA's final size has been reached. By an unknown mechanism, HA biosynthesis terminates and the polymer can be released into the extracellular space. It is also possible that HAS remains associated with the completed HA chain for some time, thus acting as an anchor on the cell surface. However, this behavior would block further production of new HA chains by the same enzyme molecule. Thus, a more likely scenario is that matrix proteins tie up the HA polysaccharide in the extracellular space to limit diffusion (Day, A.J. and Prestwich, G.D. 2002).

Features of the Pasteurella HASs: In another HAS surprise, a totally different enzyme was identified in another bacterial pathogen in 1998, the Gram-negative *Pasteurella multocida*, PmHAS (encoded by the *hyaD* gene) (DeAngelis, P.L., Jing, W., et al. 1998). PmHAS has a primary sequence dissimilar from streptococcal, vertebrate, or viral HASs. Based on sequence analysis and mutagenesis, PmHAS possesses two GT-2 domains, one for each sugar unit that is transferred (Jing, W. and DeAngelis, P.L. 2000, Jing, W. and DeAngelis, P.L. 2003) in contrast to the single GT-2 of the Class I enzymes. The GlcNAc-Tase and GlcA-Tase appeared to act

independently, resulting in non-processive polymerization. While no experimental 3D structures are available for PmHAS, a crystal structure for the similar *E. coli* chondroitin synthase, KfoC, was reported (Osawa, T., Sugiura, N., et al. 2009), revealing two separate domains, each with a UDP-sugar binding site, in agreement with earlier biochemical and mutagenesis studies of PmHAS (Jing, W. and DeAngelis, P.L. 2003). Radiation inactivation studies for PmHAS indicate that it functions as a monomer (Pummill, P.E., Kempner, E.S., et al. 2001).

Although the last HAS to be discovered, PmHAS was the first to be absolutely verified to operate by non-reducing end elongation (DeAngelis, P.L. 1999). In this case, the enzyme's propensity to use artificial acceptors allowed clear proof that one GT domain of PmHAS added a sugar unit to the nascent chain on the non-reducing terminus. Then a second sugar is added to the new terminus via the other GT domain, and so on.

The core catalytic region of the PmHAS enzyme (i.e., residues 1-703) is not membrane-integrated and truncated recombinant versions are water soluble (Fig. 3) (Jing, W. and DeAngelis, P.L. 2000). Instead, the PmHAS' carboxyl terminus (residues ~704-972) likely anchors the enzyme peripherally to the plasma membrane. It is important to note that PmHAS functions *in vivo* in concert with other membrane-associated enzymes to produce and secrete a lipid-linked capsular HA polymer (Whitfield, C., Wear, S.S., et al. 2020). During this process, the nascent HA polymer is attached to a membrane-bound glycolipid (Fig. 1C). The corresponding secretion machinery (called HexA, -B, -C, -D) consists of an ABC transporter (HexAB) integrated into the inner bacterial membrane, a periplasmic but inner membrane-anchored subunit (HexC), as well as an outer membrane component (HexD) with a large periplasmic domain. All of these genes are encoded in the same capsule biosynthesis locus as the HAS gene (reviewed in (Caffalette, C.A., Kuklewicz, J., et al. 2020).

It is conceivable that PmHAS interacts with the transport machinery to spatio-temporally couple HA biosynthesis with export. While the directionality of HA secretion from the interior of the cell to the extracellular capsule by HexABCD has not been established, it is possible that the transporter engages with the lipid anchor while the nascent HA chain is extended by PmHAS from the non-reducing terminus. This lipid-linked sugar, however, is not required for HA polymerization *in vitro*. From a biotechnological standpoint, the non-processive nature of PmHAS allows useful chemoenzymatic syntheses for the preparation of either defined oligosaccharides or quasi-monodisperse polysaccharides (reviewed in DeAngelis, P.L., Liu, J., and Linhardt, R.J. 2013) that are beyond the capabilities of any other currently known HAS.

An Updated Classification System: Based on biochemical and structural work outlined above, we present an updated classification system (Table I) derived from a previous HAS review in 2007 (Weigel, P.H. and DeAngelis, P.L. 2007) that includes three distinct types of HASs:

(*a*) Class I transmembrane synthases employing processive chain elongation that is subdivided into two groups, Class I-R (reducing end addition; Group A and C *Streptococci*) and Class I-NR (non-reducing end addition; vertebrates and PBCV-1 virus), and

(*b*) Class II peripheral membrane synthases exhibiting intrinsic non-processive elongation (non-reducing end addition; *Pasteurella multocida* Type A and close allies in the *Avibacterium* genus).

The major updates from the 2007 review include: vertebrate HAS are now combined with the viral HAS based on (*i*) the directionality of chain elongation and (*ii*) the use of a single GT-2 domain to polymerize two different monosaccharides. Both Class I catalysts couple HA synthesis with membrane translocation, but Class II does not. Sequence similarity among the

three HAS types is likely due to functional convergent evolution using the GT-2 domain structure as discussed in more detail later as well as in previous reviews (DeAngelis, P.L. 2002, Weigel, P.H. and DeAngelis, P.L. 2007).

Myths: Equivalence, Transport and Inhibitor

We have made great strides understanding HASs, but some theoretical misconceptions and a few details in the past literature will be very problematic if used as guidance, especially by novices to the field.

Myth 1: All HAS enzymes are the same. By definition, all HAS enzymes produce the same polysaccharide repeat unit. However, as explained above, the details of the initiation event (priming) and the directionality of extension (and thereby the reaction mechanism) differ between the three known HAS types.

Considering the available structural and biochemical data on bacterial and viral HASs, we can state that the two Class I types, I-R and I-NR are truly different. Analyses of viral HAS undoubtedly show that the enzyme functions as a monomer and elongates the non-reducing end of the HA polymer. AlphaFold2 predicted models of human HAS reveal a striking structural similarity with CvHAS, hence a similar reaction mechanism most likely applies to the vertebrate enzymes as well. In contrast, a wealth of biochemical data confirmed that streptococcal HASs elongate HA on the reducing end and may function as a homo-oligomer, thereby representing mechanistically distinct enzymes. Therefore, researchers need to keep in mind that insights gained from the bacterial enzymes cannot always be applied directly to the viral or vertebrate synthases and vice versa.

In summary, various GTs were most likely independently evolved multiple times, rather than being passed between various organisms. Therefore, the three HAS synthase types need to be considered as non-identical and caution is paramount during extrapolation of findings and paradigms.

Myth 2: The Class I enzymes require an ABC transporter to secrete HA to make a capsule or matrix. The hypothesis that an ABC transporter is required for Class I-enzyme mediated HA secretion (Ouskova, G., Spellerberg, B., et al. 2004, Prehm, P. and Schumacher, U. 2004) seems to have been put to rest. The discoverers of the streptococcal enzyme initially proposed that an intrinsic pore would form in the membrane based on the assumptions that (*i*) only a single *hasA* gene encoding a protein with transmembrane segments was needed to confer HA biosynthesis to hosts that lacked the HA synthases (Heldermon, C., DeAngelis, P.L., et al. 2001) and (*ii*) the HAS enzyme activity (either native or recombinant derived enzyme) *in vitro* was dependent on lipids (Triscott, M.X. and Vanderijn, I. 1986).

The ultimate proof of a coupled synthesis and translocation reaction came from *in vitro* studies on purified SeHAS (Hubbard, C., McNamara, J., et al. 2012). It was demonstrated that SeHAS, when reconstituted into proteoliposomes and exposed to substrates added to the 'outside' of the vesicles, produced HA that accumulated inside the vesicles. In the absence of any other proteinaceous components, these experiments demonstrated (a) that SeHAS is necessary and sufficient for HA synthesis and translocation, and (b) no external energy source (such as ATP or the proton motive force) is necessary for HA secretion.

Myth 3: 4-Methylumbelliferone (4-MU) is a "specific HAS inhibitor." The major mechanism of action for 4-MU is the depletion of the cytoplasmic pools of UDP-sugar donors of HAS. The exogenously added 4-MU is glucuronidated instead of the monosaccharide being attached to

UDP (Kakizaki, I., Kojima, K., et al. 2004), thereby depleting one of HAS' substrates ('no precursors, no polymer'). A direct effect on the action of the HAS has not been shown. Therefore, alternate terminology for 4-MU may be "HA biosynthesis inhibitor" or "cytoplasmic UDP-GlcA metabolic suppressor." The heparan sulfate/heparin and chondroitin sulfate GAGs are made in the Golgi, with specific nucleotide-sugar transporters ensuring substrate availability inside the organelle. Accordingly, their biosynthesis levels have not been reported to be appreciably affected by 4-MU. Furthermore, while 4-MU is an approved drug in some countries for biliary disease, its use as a HA suppressing agent for other diseases such as cancer must recognize that the reagent will have pleiotropic, off-target effects as well; recent reports indicate various perturbations that can occur (Karousou, E., Parnigoni, A., et al. 2023, Tsitrina, A.A., Halimani, N., et al. 2023). At this point, no selective potent HAS inhibitors have been reported, but perhaps future *in silico* studies or library screens with HASs will prove fruitful for leads towards a useful laboratory reagent or novel therapeutic.

Biotechnology: *In vivo* Recombinant HA Production Systems

The various HA synthases have been harnessed to yield two types of recombinant polymer production systems based on either: *a*) *in vivo* growth of engineered cells with HAS genes, or *b*) *in vitro* chemoenzymatic syntheses with purified HAS enzymes (not discussed here, but reviewed in DeAngelis, P.L., Liu, J., and Linhardt, R.J. 2013). In general, *in vivo* systems are used to produce large amounts of HA in safer, more efficient hosts.

For most microbial hosts, in addition to HAS, only one other cytosolic enzyme, UDP-glucose-6-dehydrogenase (converts UDP-glucose to UDP-GlcA) is required for HA polymerization. All eubacteria make the other needed substrate, UDP-GlcNAc, for their cell walls, thus it is typically available in sufficient quantities. Gram-positive microbes are often

preferred as they do not make endotoxin, a problematic contaminant for medical devices and medicines. For metabolic engineering beyond direct HAS gene manipulation, two major strategies to increase HA production levels are (a) to increase levels of the required donors by altering the promoters and the catalysts of the UDP-sugar synthesis pathways, and (b) to inactivate other non-HA metabolic pathways to avoid funneling off the donor sugars to non-target products. However, empirical testing is needed as the bioengineering results are not always predictable.

The streptococcal HAS was first employed in an industrial host, *Bacillus subtilis*, a Gram-positive microbe with GRAS (generally regarded as safe) status in chemically defined media (Widner, B., Behr, R., et al. 2005). Numerous other heterologous host species, including a range of bacteria, yeast and plants (Chahuki, F.F., Aminzadeh, S., et al. 2019, Cheng, F.Y., Gong, Q.Y., et al. 2016, de Oliveira, J.D., Carvalho, L.S., et al. 2016, Nazeri, A., Niazi, A., et al. 2021, Yoshimura, T., Shibata, N., et al. 2015) have since been employed. Yields were increased by manipulating the streptococcal HAS expression system (e.g., promoters, codon optimization, mutants) as well as the host's metabolic pathways (Cerminati, S., Leroux, M., et al. 2021, DeAngelis, P.L. 2012) (Yu, H. and Stephanopoulos, G. 2008) and membranes (Westbrook, A.W., Ren, X., et al. 2018). A study focused on the SeHAS enzyme's carboxyl terminus (predicted to be located inside the cytoplasm) altered HA product size (Yang, J., Cheng, F., et al. 2017); it was hypothesized that this region could be involved in chain binding/retention, thus altering processivity. This result potentially supports a model for size control based on relative affinities of the synthase for HA where weaker binding yields lower molecular weight (MW) chains and stronger interactions yield higher MWs (Weigel, P.H. and DeAngelis, P.L. 2007).

The recombinant streptococcal HAS in bacterial membranes, while not truly *in vivo* production, has also been used to generate 'HA brushes' on surfaces (e.g., glass, silica) with megaDalton polymer chains (Wei, W., Faubel, J.L., et al. 2019) that are controlled by UDP-sugar or metal ion availability and stabilized by chemical cross-linking. Such brushes can be regenerated several times as well as be micro-patterned with lasers. With further refinements, such systems may be used in future biomaterials or nanoparticles, but one obstacle is that any bacterial enzyme will be immunogenic if introduced into patients.

Over the years, production systems with other HASs including vertebrate HAS isozymes and PmHAS have been reported that generate HA in various chain sizes and yields (de Oliveira, J.D., Carvalho, L.S., et al. 2016, Gomes, A.M.V., Netto, J.H.C.M., et al. 2019, Jeong, E., Shim, W.Y., et al. 2014, Jia, Y.N., Zhu, J., et al. 2013, Mao, Z.C., Shin, H.D., et al. 2009, Nazeri, A., Niazi, A., et al. 2021). However, to date, the absolute yields generally are not as high as for the streptococcal systems.

Smaller MW HA chains have been reported to exhibit different and often antagonistic biological effects compared to larger chains, establishing the need for good sources of such materials. One approach is the use of a host co-expressing hyaluronidases to produce shorter HA fragments; the initially large chains are subsequently digested by the glycosidase action (Jin, P., Kang, Z., et al. 2016). This strategy must be calibrated to obtain the desired target size, otherwise mostly di- or tetrasaccharides (the limit digest size, depending on the hyaluronidase species) are obtained.

New Frontiers and Remaining Questions

While the HAS field has made significant progress, much is still unknown and there are areas worth exploring. Some of the "new areas" posed fifteen years ago (Weigel, P.H. and

DeAngelis, P.L. 2007) remain unsolved, so hopefully in the following decades these questions will not remain.

Molecular details on the Class I HAS single domain substrate recognition/usage: A pair of glucose-based sugars, GlcNAc and GlcA, is transferred by the same HAS active site to two different ring positions on the nascent HA chain. How does this catalytic center make the repeating HA chain with the observed high fidelity? Some crosstalk between HA's terminal glycosyl unit and the incoming substrate must exist to ensure (a) alternating polymerization of GlcNAc and GlcA and (b) regio- and stereo-selective glycosyl transfer. Once this is better understood and if the catalyst is malleable, perhaps the creation of novel designer polysaccharides beyond HA with artificial sugar units and/or linkages will be possible.

Explanation of Class II efficiency: PmHAS is a non-processive, yet speedy elongation catalyst based on observations that the wild-type bifunctional enzyme had the same efficiency *in vitro* as a pair of mutant catalysts with either single-action GlcNAc-Tase or GlcA-Tase activity (Jing, W. and DeAngelis, P.L. 2000). Is this simply kinetic optimization with the substrates or do guide elements (e.g., molecular steering, surface channels) assist the non-reducing terminus transiting between the two GT sites? We know that the optimal acceptors *in vitro* possess 3 to 4 sugar units (Williams, K.J., Halkes, K.M., et al. 2006) and assume that the binding site size is comparable, but beyond that, little is known.

Polymer size control: While initiation and elongation has been partly explained, the ultimate control of chain length by the intrinsic properties of the Class I HAS enzymes (not by the simple control of substrate UDP-GlcA levels) observed *in vitro* as well as in the living cell is still unsolved. Several hypotheses were presented in 2007 (Weigel, P.H. and DeAngelis, P.L. 2007) and none have been either substantially strengthened or diminished since then in a

coherent, comprehensive fashion. Likewise, the nature of HA size control for the Class II PmHAS *in vivo* is not known yet; is the HAS itself or the transport system or both components responsible?

Post-translational modification and trafficking of Class I-NR HASs: Several reports of mammalian HAS modification (e.g., phosphorylation, glycosylation) have been made (Caon, I., Parnigoni, A., et al. 2021, de Mera, R.M.M.F., Arasu, U.T., et al. 2019, Kasai, K., Kuroda, Y., et al. 2020, Vigetti, D. and Passi, A. 2014). However, the functional consequences of these modifications in modulating catalysis, turnover, and/or intracellular transport are not yet completely defined. The modifications appear to be important based on site-specific mutagenesis experiments. Perhaps the combined use of more defined *in vitro* systems, selective modification inhibitors, and/or fluorophore-tagged HAS probes *in vivo* will yield better mechanistic insights in the next decade.

How is HA biosynthesis controlled during vesicular trafficking? HA does not appear to significantly accumulate in the ER or Golgi vesicles; if it did so, then the bulky polymer most likely would impair the organelles' functions. Hence, how is HA biosynthesis confined to the plasma membrane? Perhaps some post-translational modification(s) transiently silence HAS during vesicular trafficking.

Confirmation of other HAS candidates: Based on sequence homologies to the rigorously verified HASs discussed above, other enzyme candidates have been reported. A protein found in some strains of the fungal pathogen *Cryptococcus neoformans*, CPS1, resembles the *S. pneumoniae* Cps3S capsular synthase (~26 % identity at protein sequence level) and to a slightly lesser degree, the streptococcal HASs. The cryptococcal CPS1 is quite likely to be an authentic HAS based on the reported biochemical tests (e.g., binding by versican, FACE disaccharide gels)

(Jong, A., Wu, C.H., et al. 2007), but further analysis of the polysaccharide product (especially using the HA-specific *Streptomyces* hyaluronidase and/or NMR) is needed to truly confirm the polysaccharide structure.

In another case, a gene on the pXO1 plasmid in certain virulent *Bacillus anthracis* strains (e.g., Sterne) was noted for potentially encoding a HAS based on its close similarity to the streptococcal HASs (~52% identity at protein sequence level) (Okinaka, R.T., Cloud, K., et al. 1999). This causative agent of anthrax employs toxins (also encoded on the pXO1 plasmid) and a poly-glutamic acid capsule (encoded on another plasmid) as virulence factors. This SpHAS-like gene has not been authenticated since its initial report, but this is probably due in part to a predicted inactivating frameshift mutation. Later, some very pathogenic strains of *B. cereus*, a species related to the anthrax pathogen, were found to possess a similar plasmid to pXO1 that also encoded a very similar HAS candidate (~99% identical to the *B. anthracis* gene) located` in an operon with UDP-glucose dehydrogenase and UDP-glucose pyrophosphorylase like the Group A and C *Streptococci*, but with a different gene order (Oh, S., Budzik, J., et al. 2011). The large capsule in these *B. cereus* strains was removed by treatment with testicular hyaluronidase or by knockout of the HAS-like gene. Again, more specific tools and/or biophysical analyses will be needed to confirm this polysaccharide's structure as HA.

In closing, the various HA synthases promise to be exciting subjects for both basic and applied sciences if we remain cognizant of their enzymological similarities and differences as well as stay observant for any new features or behaviors that are sure to emerge.

Funding: Work by PD was supported by federal (NIH, NSF) and state agencies (Oklahoma Center for Advancement of Science and Technology) and the Presbyterian Health Foundation. JZ is an Investigator of the Howard Hughes Medical Institute and acknowledges NIH funding for HA research.

Acknowledgments: We thank our students and colleagues in the field for their thoughts and contributions over the years. We also thank: Dixy Green for manuscript review, Bernard Henrissatt for discussions on the CAZy enzyme database, James van Etten for thoughts on *Chlorella* virus evolution, and Chris Whitfield for discussions on polysaccharide transport. Meetings by the Society for Glycobiology, International Society for Hyaluronan Sciences (ISHAS), and FASEB Microbial Glycobiology played instrumental roles as crucibles to help amalgamate a wide range of data into new insights and directions.

Abbreviations: AF, AlphaFold2; GlcA, glucuronic acid; GlcNAc, N-acetylglucosamine; GT, glycosyltransferase, HA, hyaluronan, hyaluronic acid, hyaluronate; HAS, HA synthase; 4-MU, 4-methylumbelliferone; MW, molecular weight; NR non-reducing; R, reducing; UDP, uridine diphosphate

Data Availability Statement: Alphafold HAS structures available upon request.

References

Almond A, DeAngelis PL, Blundell CD. 2006. Hyaluronan: the local solution conformation determined by NMR and computer modeling is close to a contracted left-handed 4-fold helix. *J Mol Biol*, 358:1256-1269.

Banerji S, Wright AJ, Noble M, Mahoney DJ, Campbell ID, Day AJ, Jackson DG. 2007. Structures of the CD44-hyaluronan complex provide insight into a fundamental carbohydrate-protein interaction. *Nat Struct Mol Biol*, 14:234-239.

Bart G, Vico NO, Hassinen A, Pujol FM, Deen AJ, Ruusala A, Tammi RH, Squire A, Heldin P, Kellokumpu S*, et al.* 2015. Fluorescence resonance energy transfer (FRET) and proximity ligation assays reveal functionally relevant homo- and heteromeric complexes among hyaluronan synthases HAS1, HAS2, and HAS3. *J Biol Chem*, 290:11479-11490.

Blackburn MR, Hubbard C, Kiessling V, Bi Y, Kloss B, Tamm LK, Zimmer J. 2018. Distinct reaction mechanisms for hyaluronan biosynthesis in different kingdoms of life. *Glycobiol*, 28:108-121.

Bodevin-Authelet S, Kusche-Gullberg M, Pummill PE, DeAngelis PL, Lindahl U. 2005. Biosynthesis of hyaluronan: direction of chain elongation. *J Biol Chem*, 280:8813-8818.

Caffalette CA, Kuklewicz J, Spellmon N, Zimmer J. 2020. Biosynthesis and Export of Bacterial Glycolipids. *Annu Rev Biochem*, 89:741-768.

Caon I, Parnigoni A, Viola M, Karousou E, Passi A, Vigetti D. 2021. Cell Energy Metabolism and Hyaluronan Synthesis. *Journal of Histochemistry & Cytochemistry*, 69:35-47.

Carpita NC. 2011. Update on mechanisms of plant cell wall biosynthesis: how plants make cellulose and other (1->4)-beta-D-glycans. *Plant Physiol*, 155:171-184.

Cerminati S, Leroux M, Anselmi P, Peiru S, Alonso JC, Priem B, Menzella HG. 2021. Low cost and sustainable hyaluronic acid production in a manufacturing platform based on *Bacillus subtilis* 3NA strain (vol 105, pg 3075, 2021). *Applied Microbiology and Biotechnology*, 105:6529-6529.

Chahuki FF, Aminzadeh S, Jafarian V, Tabandeh F, Khodabandeh M. 2019. Hyaluronic acid production enhancement via genetically modification and culture medium optimization in *Lactobacillus acidophilus*. *International Journal of Biological Macromolecules*, 121:870-881.

Cheng FY, Gong QY, Yu HM, Stephanopoulos G. 2016. High-titer biosynthesis of hyaluronic acid by recombinant *Corynebacterium glutamicum*. *Biotechnology Journal*, 11:574-584.

Day AJ, Prestwich GD. 2002. Hyaluronan-binding proteins: tying up the giant. *J Biol Chem*, 277:4585-4588.

de Mera RMMF, Arasu UT, Karna R, Oikari S, Rilla K, Vigetti D, Passi A, Heldin P, Tammi MI, Deen AJ. 2019. Effects of mutations in the post-translational modification sites on the trafficking of hyaluronan synthase 2 (HAS2). *Matrix Biology*, 80:85-103.

de Oliveira JD, Carvalho LS, Gomes AMV, Queiroz LR, Magalhaes BS, Parachin NS. 2016. Genetic basis for hyper production of hyaluronic acid in natural and engineered microorganisms. *Microbial Cell Factories*, 15.

DeAngelis PL. 1999. Hyaluronan synthases: fascinating glycosyltransferases from vertebrates, bacterial pathogens, and algal viruses. *Cell Mol Life Sci*, 56:670-682.

DeAngelis PL, Jing W, Graves M, Burbank D, Van Etten J. 1997. Hyaluronan synthase of chlorella virus PBCV-1. *Science*, 278:1800-1803.

DeAngelis PL. 1999. Molecular directionality of polysaccharide polymerization by the Pasteurella multocida hyaluronan synthase. *J Biol Chem*, 274:26557-26562.

DeAngelis PL. 2002. Microbial glycosaminoglycan glycosyltransferases. *Glycobiology*, 12:9R-16R.

DeAngelis PL. 2012. Glycosaminoglycan polysaccharide biosynthesis and production: today and tomorrow. *Applied Microbiology and Biotechnology*, 94:295-305.

DeAngelis PL, Achyuthan AM. 1996. Yeast-derived recombinant DG42 protein of *Xenopus* can synthesize hyaluronan in vitro. *J Biol Chem*, 271:23657-23660.

DeAngelis PL, Jing W, Drake RR, Achyuthan AM. 1998. Identification and molecular cloning of a unique hyaluronan synthase from *Pasteurella multocida*. *J Biol Chem*, 273:8454-8458.

DeAngelis PL, Papaconstantinou J, Weigel PH. 1993. Molecular cloning, identification, and sequence of the hyaluronan synthase gene from group A *Streptococcus pyogenes*. *J Biol Chem*, 268:19181-19184.

Gomes AMV, Netto JHCM, Carvalho LS, Parachin NS. 2019. Heterologous Hyaluronic Acid Production in *Kluyveromyces lactis*. *Microorganisms*, 7.

Guan L, Zhang L, Xue Y, Yang J, Zhao Z. 2020. Molecular pathogenesis of the hyaluronic acid capsule of *Pasteurella multocida*. *Microb Pathog*, 149:104380.

Heldermon C, DeAngelis PL, Weigel PH. 2001. Topological organization of the hyaluronan synthase from *Streptococcus pyogenes*. *J Biol Chem*, 276:2037-2046.

Hoshi H, Nakagawa H, Nishiguchi S, Iwata K, Niikura K, Monde K, Nishimura S. 2004. An engineered hyaluronan synthase: characterization for recombinant human hyaluronan synthase 2 *Escherichia coli*. *J Biol Chem*, 279:2341-2349.

Hubbard C, McNamara J, Azumaya C, Patel M, Zimmer J. 2012. The hyaluronan synthase catalyzes the synthesis and membrane translocation of hyaluronan. *J Mol Biol*, 418:21-31.

Itano N, Kimata K. 1996. Expression cloning and molecular characterization of HAS protein, a eukaryotic hyaluronan synthase. *Journal of Biological Chemistry*, 271:9875-9878.

Itano N, Zhuo L, Kimata K. 2008. Impact of the hyaluronan-rich tumor microenvironment on cancer initiation and progression. *Cancer Sci*, 99:1720-1725.

Jeong E, Shim WY, Kim JH. 2014. Metabolic engineering of *Pichia pastoris* for production of hyaluronic acid with high molecular weight. *J Biotechnol*, 185:28-36.

Jia YN, Zhu J, Chen XF, Tang DY, Su D, Yao WB, Gao XD. 2013. Metabolic engineering of *Bacillus subtilis* for the efficient biosynthesis of uniform hyaluronic acid with controlled molecular weights. *Bioresource Technology*, 132:427-431.

Jin P, Kang Z, Yuan PH, Du GC, Chen J. 2016. Production of specific-molecular-weight hyaluronan by metabolically engineered *Bacillus subtilis* 168. *Metabolic Engineering*, 35:21-30.

Jing W, DeAngelis PL. 2000. Dissection of the two transferase activities of the *Pasteurella multocida* hyaluronan synthase: two active sites exist in one polypeptide. *Glycobiology*, 10:883-889.

Jing W, DeAngelis PL. 2003. Analysis of the two active sites of the hyaluronan synthase and the chondroitin synthase of *Pasteurella multocida*. *Glycobiology*, 13:661-671.

Jong A, Wu CH, Chen HM, Luo F, Kwon-Chung KJ, Chang YC, Lamunyon CW, Plaas A, Huang SH. 2007. Identification and characterization of CPS1 as a hyaluronic acid synthase contributing to the pathogenesis of *Cryptococcus neoformans* infection. *Eukaryot Cell*, 6:1486-1496.

Kakizaki I, Kojima K, Takagaki K, Endo M, Kannagi R, Ito M, Maruo Y, Sato H, Yasuda T, Mita S*, et al.* 2004. A novel mechanism for the inhibition of hyaluronan biosynthesis by 4-methylumbelliferone. *Journal of Biological Chemistry*, 279:33281-33289.

Karousou E, Parnigoni A, Moretto P, Passi A, Viola M, Vigetti D. 2023. Hyaluronan in the Cancer Cells Microenvironment. *Cancers*, 15.

Kasai K, Kuroda Y, Takabuchi Y, Nitta A, Kobayashi T, Nozaka H, Miura T, Nakamura T. 2020. Phosphorylation of Thr(328) in hyaluronan synthase 2 is essential for hyaluronan synthesis. *Biochem Biophys Res Commun*, 533:732-738.

Kendall FE, Heidelberger M, Dawson MH. 1937. A serologically inactive polysaccharide elaborated by mucoid strains of group a hemolytic streptococcus. *Journal of Biological Chemistry*, 118:61-69.

Kumari K, Weigel PH. 1997. Molecular cloning, expression, and characterization of the authentic hyaluronan synthase from group C *Streptococcus equisimilis*. *J Biol Chem*, 272:32539-32546.

Lansing M, Lellig S, Mausolf A, Martini I, Crescenzi F, O'Regan M, Prehm P. 1993. Hyaluronate synthase: cloning and sequencing of the gene from *Streptococcus sp*. *Biochem J*, 289 (Pt 1):179-184.

Lombard V, Ramulu HG, Drula E, Coutinho PM, Henrissat B. 2014. The carbohydrate-active enzymes database (CAZy) in 2013. *Nucleic Acids Res*, 42:D490-D495.

Maloney FP, Kuklewicz J, Corey RA, Bi YC, Ho RY, Mateusiak L, Pardon E, Steyaert J, Stansfeld PJ, Zimmer J. 2022. Structure, substrate recognition and initiation of hyaluronan synthase. *Nature*, 604:195-201.

Mao ZC, Shin HD, Chen R. 2009. A recombinant *E. coli* bioprocess for hyaluronan synthesis. *Applied Microbiology and Biotechnology*, 84:63-69.

Markovitz A, Cifonelli JA, Dorfman A. 1959. The biosynthesis of hyaluronic acid by group A *Streptococcus*. VI. Biosynthesis from uridine nucleotides in cell-free extracts. *J Biol Chem*, 234:2343-2350.

Meyer K, Palmer J. 1936. The polysaccharide of the vitreous humo. *J Biol Chem.*, 107:629-634.

Meyer MF, Kreil G. 1996. Cells expressing the DG42 gene from early *Xenopus* embryos synthesize hyaluronan. *Proc Natl Acad Sci U S A*, 93:4543-4547.

Mian N. 1986. Characterization of a high-Mr plasma-membrane-bound protein and assessment of its role as a constituent of hyaluronate synthase complex. *Biochem J*, 237:343-357.

Morgan JL, McNamara JT, Fischer M, Rich J, Chen HM, Withers SG, Zimmer J. 2016. Observing cellulose biosynthesis and membrane translocation *in crystallo*. *Nature*, 531:329-334.

Nazeri A, Niazi A, Afsharifar A, Taghavi SM, Moghadam A, Aram F. 2021. Heterologous production of hyaluronic acid in *Nicotiana tabacum* hairy roots expressing a human hyaluronan synthase 2. *Scientific Reports*, 11.

Oh S, Budzik J, Garufi G, Schneewind O. 2011. Two capsular polysaccharides enable *Bacillus cereus* G9241 to cause anthrax-like disease. *Mol Microbiol*, 80:455-470.

Okinaka RT, Cloud K, Hampton O, Hoffmaster AR, Hill KK, Keim P, Koehler TM, Lamke G, Kumano S, Mahillon J*, et al.* 1999. Sequence and organization of pXO1, the large *Bacillus anthracis* plasmid harboring the anthrax toxin genes. *Journal of Bacteriology*, 181:6509-6515.

Omadjela O, Narahari A, Strumillo J, Mélida H, Mazur O, Bulone V, Zimmer J. 2013. BcsA and BcsB form the catalytically active core of bacterial cellulose synthase sufficient for *in vitro* cellulose synthesis. *Proc Natl Acad Sci U S A*, 110:17856-17861.

Osawa T, Sugiura N, Shimada H, Hirooka R, Tsuji A, Shirakawa T, Fukuyama K, Kimura M, Kimata K, Kakuta Y. 2009. Crystal structure of chondroitin polymerase from *Escherichia coli* K4. *Biochemical and Biophysical Research Communications*, 378:10-14.

Ouskova G, Spellerberg B, Prehm P. 2004. Hyaluronan release from *Streptococcus pyogenes*: export by an ABC transporter. *Glycobiology*, 14:931-938.
Pedersen LC, Tsuchida K, Kitagawa H, Sugahara K, Darden TA, Negishi M. 2000. Heparan/chondroitin sulfate biosynthesis. Structure and mechanism of human glucuronyltransferase I. *J Biol Chem*, 275:34580-34585.
Prehm P. 1989. Identification and regulation of the eukaryotic hyaluronate synthase. *Ciba Found Symp*, 143:21-30; discussion 30-40, 281-285.
Prehm P, Schumacher U. 2004. Inhibition of hyaluronan export from human fibroblasts by inhibitors of multidrug resistance transporters. *Biochemical pharmacology*, 68:1401-1410.
Pummill PE, Achyuthan AM, DeAngelis PL. 1998. Enzymological characterization of recombinant *Xenopus* DG42, a vertebrate hyaluronan synthase. *J Biol Chem*, 273:4976-4981.
Pummill PE, Kempner ES, DeAngelis PL. 2001. Functional molecular mass of a vertebrate hyaluronan synthase as determined by radiation inactivation analysis. *Journal of Biological Chemistry*, 276:39832-39835.
Purushotham P, Cho SH, Diaz-Moreno SM, Kumar M, Nixon BT, Bulone V, Zimmer J. 2016. A single heterologously expressed plant cellulose synthase isoform is sufficient for cellulose microfibril formation *in vitro*. *Proc Natl Acad Sci U S A*, 113:11360-11365.
Purushotham P, Ho R, Zimmer J. 2020. Architecture of a catalytically active homotrimeric plant cellulose synthase complex. *Science* 369:1089-1094.
Sargent TD, Jamrich M, Dawid IB. 1986. Cell interactions and the control of gene activity during early development of *Xenopus laevis*. *Dev Biol*, 114:238-246.
Saxena IM, Brown RM, Dandekar T. 2001. Structure--function characterization of cellulose synthase: relationship to other glycosyltransferases. *Phytochemistry*, 57:1135-1148.
Simpson M, Schaefer L, Hascall V, Esko JD. 2022. Hyaluronan. In: Varki A, Cummings RD, Esko JD, Stanley P, Hart GW, Aebi M, Mohnen D, Kinoshita T, Packer NH, Prestegard JH, et al. editors. *Essentials of Glycobiology*. Cold Spring Harbor (NY). p. 205-216.
Spicer AP, Augustine ML, McDonald JA. 1996. Molecular cloning and characterization of a putative mouse hyaluronan synthase. *J Biol Chem*, 271:23400-23406.
Tammi MI, Day AJ, Turley EA. 2002. Hyaluronan and homeostasis: a balancing act. *J Biol Chem*, 277:4581-4584.
Tlapak-Simmons VL, Baron CA, Gotschall R, Haque D, Canfield WM, Weigel PH. 2005. Hyaluronan biosynthesis by class I streptococcal hyaluronan synthases occurs at the reducing end. *J Biol Chem*, 280:13012-13018.
Tlapak-Simmons VL, Baron CA, Weigel PH. 2004. Characterization of the purified hyaluronan synthase from *Streptococcus equisimilis*. *Biochemistry*, 43:9234-9242.
Tlapak-Simmons VL, Kempner ES, Baggenstoss BA, Weigel PH. 1998. The active streptococcal hyaluronan synthases (HASs) contain a single HAS monomer and multiple cardiolipin molecules. *J Biol Chem*, 273:26100-26109.
Toole BP. 2000. Hyaluronan is not just a goo! *J Clin Invest*, 106:335-336.
Triscott MX, Vanderijn I. 1986. Solubilization of Hyaluronic-Acid Synthetic Activity from Streptococci and Its Activation with Phospholipids. *Journal of Biological Chemistry*, 261:6004-6009.
Tsitrina AA, Halimani N, Andreichenko IN, Sabirov M, Nesterchuk M, Dashenkova NO, Romanov R, Bulgakova EV, Mikaelyan A, Kotelevtsev Y. 2023. 4-Methylumbelliferone Targets Revealed by Public Data Analysis and Liver Transcriptome Sequencing. *International Journal of Molecular Sciences*, 24.

van de Rijn I, Drake RR. 1992. Analysis of the streptococcal hyaluronic acid synthase complex using the photoaffinity probe 5-azido-UDP-glucuronic acid. *The Journal of biological chemistry*, 267:24302-24306.

Varki A. 1996. Does DG42 synthesize hyaluronan or chitin?: A controversy about oligosaccharides in vertebrate development. *Proceedings of the National Academy of Sciences of the United States of America*, 93:4523-4525.

Vigetti D, Passi A. 2014. Hyaluronan synthases posttranslational regulation in cancer. *Advances in cancer research*, 123:95-119.

Wei W, Faubel JL, Selvakumar H, Kovari DT, Tsao J, Rivas F, Mohabir AT, Krecker M, Rahbar E, Hall AR*, et al.* 2019. Self-regenerating giant hyaluronan polymer brushes. *Nature communications*, 10:5527.

Weigel PH. 2015. Hyaluronan Synthase: The Mechanism of Initiation at the Reducing End and a Pendulum Model for Polysaccharide Translocation to the Cell Exterior. *Int J Cell Biol*, 2015:367579.

Weigel PH, DeAngelis PL. 2007. Hyaluronan synthases: a decade-plus of novel glycosyltransferases. *J Biol Chem*, 282:36777-36781.

Weigel PH, Hascall VC, Tammi M. 1997. Hyaluronan synthases. *J Biol Chem*, 272:13997-14000.

Weigel PH, West CM, Zhao P, Wells L, Baggenstoss BA, Washburn JL. 2015. Hyaluronan synthase assembles chitin oligomers with -GlcNAc(alpha1->)UDP at the reducing end. *Glycobiology*.

Wessels MR. 2019. Capsular Polysaccharide of Group A *Streptococcus*. *Microbiol Spectr*, 7.

Westbrook AW, Ren X, Moo-Young M, Chou CP. 2018. Engineering of cell membrane to enhance heterologous production of hyaluronic acid in *Bacillus subtilis*. *Biotechnology and Bioengineering*, 115:216-231.

Whitfield C, Wear SS, Sande C. 2020. Assembly of Bacterial Capsular Polysaccharides and Exopolysaccharides. *Annual Review of Microbiology*, 74:521-543.

Widner B, Behr R, Von Dollen S, Tang M, Heu T, Sloma A, Sternberg D, DeAngelis PL, Weigel PH, Brown S. 2005. Hyaluronic acid production in *Bacillus subtilis*. *Applied and Environmental Microbiology*, 71:3747-3752.

Williams KJ, Halkes KM, Kamerling JP, DeAngelis PL. 2006. Critical elements of oligosaccharide acceptor substrates for the *Pasteurella multocida* hyaluronan synthase. *Journal of Biological Chemistry*, 281:5391-5397.

Yang J, Cheng F, Yu H, Wang J, Guo Z, Stephanopoulos G. 2017. Key Role of the Carboxyl Terminus of Hyaluronan Synthase in Processive Synthesis and Size Control of Hyaluronic Acid Polymers. *Biomacromolecules*, 18:1064-1073.

Yoshimura T, Shibata N, Hamano Y, Yamanaka K. 2015. Heterologous Production of Hyaluronic Acid in an epsilon-Poly-L-Lysine Producer, *Streptomyces albulus*. *Appl Environ Microbiol*, 81:3631-3640.

Yu H, Stephanopoulos G. 2008. Metabolic engineering of *Escherichia coli* for biosynthesis of hyaluronic acid. *Metab Eng*, 10:24-32.

Zimmer BM, Barycki JJ, Simpson MA. 2022. Mechanisms of coordinating hyaluronan and glycosaminoglycan production by nucleotide sugars. *Am J Physiol Cell Physiol*, 322:C1201-C1213.

Figure Legends

Figure 1. **Schematic of HA matrix, polymer structure and synthases. A**: Eukaryotic cells have an HA polysaccharide coating (along with various proteins, not shown) on their extracellular surfaces (HA matrix and chain, *blue*) while select microbial cells have an HA-rich capsule. **B**. HA polysaccharide has a $[\text{-3-GlcNAc-1-}\beta\text{-4-GlcA-1-}\beta\text{-}]_n$ repeating structure where *n* can range up to 10^4. **C**. Depending on the species, one of three types of HA synthase enzymes (*see* Table I) polymerizes the monosaccharides from UDP-sugar donors into the HA chain that is then secreted or transported into the extracellular space. Class II HASs synthesize HA *in vivo* on a glycolipid anchor (indicated by a lipid-linked *white square*) generated by enzymes encoded in the corresponding capsular polysaccharide biosynthesis gene operon.

Figure 2. **Schematic models of HA biosynthesis past and present. A**: The first reported model of the formation of HA biosynthesis (adapted from Markovitz A., Cifonelli J.A., Dorfman A., 1959; reproduced with permission from *J. Biol. Chem.*) where alternating attack by incoming UDP-sugars results in the formation of sugar repeats. **B.** Modern models of the two types of Class I HAS; while both have channels to secrete the identical HA chain, the two types differ in molecular directionality of chain elongation. For I-R synthases, the monosaccharide of the UDP-donor attacks the reducing end of the nascent UDP-HA chain intermediate; these enzymes have been proposed to dimerize to simultaneously bind UDP-linked HA and donor sugars. On the other hand, for I-NR HASs, the terminal monosaccharide at the non-reducing end of the nascent HA chain attacks the UDP-sugar donor.

Figure 3. **Structural models of the various HASs**. The experimentally determined structure of CvHAS (PDB: 7SPA; Class I-NR) is shown as a cartoon with TM helices colored in *gray*, interface helices in *blue*, and the catalytic GT-2 domain in *magenta* and *cyan* for beta-strands and helices, respectively. The priming GlcNAc monosaccharide is shown with *orange spheres* for carbon atoms. AlphaFold2 (AF) models are shown for HAS from *Homo sapiens* (Hs; Class I-NR), *Streptococcus equisimilis* (Se; Class I-R), and *Pasteurella multocida* (Pm; Class II). The putative HA translocation pathways are shown as a *blue dashed line*. SeHAS is likely to form an HA translocation channel at a dimer interface (the second HAS polypeptide is shown in *light blue* to the left 3D structure), but further work is needed to confirm. Aspartate residues (the first Asp in D-X-D motif) implicated in substrate binding are shown and labeled for all species. The indicated Trp (W) residues mark the acceptor position above the Class I catalytic pockets.

Figure 4. **Model of HA biosynthesis by Class I-NR enzymes**. The postulated steps of polysaccharide initiation and production in order from left to right. HAS generates a monosaccharide primer by hydrolyzing a UDP-GlcNAc substrate. Substrate selectivity is determined by the nature of the accepting glycosyl unit. GlcNAc's acetamido group prevents binding of a UDP-GlcNAc substrate and a GlcA acceptor is incompatible with binding of a UDP-GlcA substrate. The control of HA chain elongation and termination is not yet understood.

	Class I		Class II
	I-R	I-NR	
	**SpHAS, SeHAS (& allies; CPS1, BcHAS)**	**CvHAS (XlHAS, MmHAS, HsHAS isozymes)**	**PmHAS (& allies)**
**Number of GT-2 modules (UDP-sugar binding sites)**	One	One	Two
**Predicted membrane topology**	Integral	Integral	Peripheral (soluble)
**Intrinsic transmembrane channel**	Yes	Yes	No
**HA chain growth directionality**	Reducing	Non-reducing	Non-reducing
**Intrinsic polymerization mode**	Processive	Processive	Non-processive

TABLE I. Updated HA synthase classification system.

The various known HASs have distinct biochemical and structural characteristics classified into three distinct types. The *parentheses* indicate HASs that have not been definitively confirmed, but are quite likely to be grouped in these classes. **Class I**: (*i*) the same GT domain (CAZY GT-2) adds two different monosaccharide units, and (*ii*) possess a transmembrane channel for HA secretion. This class is subdivided based on chain elongation directionality into either: **I-R**: addition of incoming sugar to reducing end, or **I-NR**: addition of incoming sugar to non-reducing end. **Class II**: two fused, but independent GT-2 domains; no intrinsic transport functionality. (Sp, *Streptococcus pyogenes;* Se*, S. equisimilis*; Cv, *Chlorella* virus PBCV-1; Xl, *Xenopus laevis*, frog; Mm, *Mus musculus,* mouse; Hs, *Homo sapiens*, human; Pm, *Pasteurella multocida;* CPS1, HAS candidate from *Cryptococcus neoformans*; BcHAS, *hasA* candidate from *Bacillus cereus*)
